# Can we derive an 'exchange rate' between descriptive and preference-based outcome measures for stroke? Results from the transfer to utility (TTU) technique

**DOI:** 10.1186/1477-7525-7-33

**Published:** 2009-04-17

**Authors:** Duncan Mortimer, Leonie Segal, Jonathan Sturm

**Affiliations:** 1 Centre for Health Economics, Monash University, Building 75, The Strip, Clayton 3800, Australia; 2 Division of Health Sciences, University of South Australia, Adelaide 5000, Australia; 3 Department of Neurology, Gosford Hospital, PO Box 361, New South Wales 2250, Australia

## Abstract

**Background:**

Stroke-specific outcome measures and descriptive measures of health-related quality of life (HRQoL) are unsuitable for informing decision-makers of the broader consequences of increasing or decreasing funding for stroke interventions. The quality-adjusted life year (QALY) provides a common metric for comparing interventions over multiple dimensions of HRQoL and mortality differentials. There are, however, many circumstances when – because of timing, lack of foresight or cost considerations – only stroke-specific or descriptive measures of health status are available and some indirect means of obtaining QALY-weights becomes necessary. In such circumstances, the use of regression-based transformations or mappings can circumvent the failure to elicit QALY-weights by allowing predicted weights to proxy for observed weights. This regression-based approach has been dubbed 'Transfer to Utility' (TTU) regression. The purpose of the present study is to demonstrate the feasibility and value of TTU regression in stroke by deriving transformations or mappings from stroke-specific and generic but descriptive measures of health status to a generic preference-based measure of HRQoL in a sample of Australians with a diagnosis of acute stroke. Findings will quantify the additional error associated with the use of condition-specific to generic transformations in stroke.

**Methods:**

We used TTU regression to derive empirical transformations from three commonly used descriptive measures of health status for stroke (NIHSS, Barthel and SF-36) to a preference-based measure (AQoL) suitable for attaching QALY-weights to stroke disease states; based on 2570 observations drawn from a sample of 859 patients with stroke.

**Results:**

Transformations from the SF-36 to the AQoL explained up to 71.5% of variation in observed AQoL scores. Differences between mean predicted and mean observed AQoL scores from the 'severity-specific' item- and subscale-based SF-36 algorithms and from the 'moderate to severe' index- and item-based Barthel algorithm were neither clinically nor statistically significant when 'low severity' SF-36 transformations were used to predict AQoL scores for patients in the NIHSS = 0 and NIHSS = 1–5 subgroups and when 'moderate to severe severity' transformations were used to predict AQoL scores for patients in the NIHSS ≥ 6 subgroup. In contrast, the difference between mean predicted and mean observed AQoL scores from the NIHSS algorithms and from the 'low severity' Barthel algorithms reached levels that could mask minimally important differences on the AQoL scale.

**Conclusion:**

While our NIHSS to AQoL transformations proved unsuitable for most applications, our findings demonstrate that stroke-relevant outcome measures such as the SF-36 and Barthel Index can be adequately transformed to preference-based measures for the purposes of economic evaluation.

## Introduction

The economic evaluation of health programs is often and increasingly a prerequisite in obtaining funding from third-party payers seeking to get the best value from a limited health budget. Where treatment is expected to impact on health-related quality of life (HRQoL), selecting an appropriate outcome measure frequently entails a trade-off between the sensitivity of available instruments for the disease or condition under study and the comparability (and therefore policy-relevance) of study results. Leaving aside the question of whether disease-specific outcome measures really are more sensitive than more generic measures, a number of difficulties arise in selecting a comparable outcome measure for use in economic evaluation.

While the minimal clinically significant improvement on a descriptive measure such as the SF-36, NIHSS or Barthel could be used to partition the trial population into responders and non-responders before expressing findings in terms of cost per additional responder, such an approach would not achieve comparability of findings even in the event that every other evaluation was also to express results in terms of responders. Because descriptive measures lack weak interval properties, there is no guarantee that a 10 point improvement at the upper end of the scale is equivalent to a 10 point improvement at the lower end of the scale. The weak interval property simply requires that a given numerical change along a scale should have the same meaning regardless of the direction and location of that change [1]. Descriptive measures such as the SF-36, NIHSS and Barthel provide an interval scale only by coincidence because items receive either an ad hoc or equal weighting when calculating subscale or dimension scores (and subscales or dimensions typically receive either an ad hoc or equal weighting when calculating scale scores). Or, as Gold et al. [2] put it, descriptive measures "assume that the number of items on each dimension provides an adequate reflection of the importance of the various domains contained in the questionnaire. ...simply summing numerical weightings across questions on a scale does not guarantee that changes in scores will coincide with changes in health status that are seen as better or worse by patients or the general public" (p97–98).

To achieve comparability across interventions and across disease-areas, cost-effectiveness analysis is increasingly eschewed in favour of cost-utility analysis with the quality adjusted life year (QALY) providing a common metric for the valuation of mortality *and *relevant dimensions of HRQoL. Richardson [1] describes the conditions under which QALY-weights can be considered to have strong and weak interval properties. Selecting a comparable outcome measure for use in economic evaluation then reduces to a choice between alternative methods of obtaining QALY-weights that reflect preferences over health states observed in the study population [2,3]. QALY-weights could, for example, be directly elicited from study participants using a preference-based scaling technique such as the time trade-off (TTO) to value their own health state, or by using a preference-based multi-attribute utility instrument such as the EQ5D to assign a 'stock' QALY-weight (obtained from another population during scaling) to questionnaire responses describing each participant's own health state [4].

There are, however, many circumstances when – because of timing, lack of foresight or cost considerations – only descriptive (rather than preference-based) measures of quality of life are available and some other means of obtaining QALY-weights becomes necessary. In such circumstances, the use of regression-based transformations or mappings can circumvent the failure to elicit QALY-weights from study participants by allowing predicted scores for preference-based measures such as the EQ5D or TTO to proxy for directly observed EQ5D or TTO scores. This regression-based approach to estimating a statistical transformation or exchange rate from a descriptive measure of HRQoL to a preference-based measure of HRQoL has been dubbed 'Transfer to Utility' (TTU) regression [5]. Given the development of a suitable regression-based transformation, TTU regression permits conversion of outcomes commonly used in clinical trials into the common metric of QALYs. While this constitutes a second best approach, it represents an extremely useful technique in the absence of the widespread use of preference-based measures in the conduct of clinical trials.

The principle underlying the TTU approach is that both descriptive and preference-based health outcome instruments estimate the effect of the intervention with respect to one or more relevant dimensions of HRQoL. To the extent that the coverage and sensitivity of the two instruments corresponds, the difference between instruments arises due to out-right errors that might be reflected in the reliability of each instrument (or lack thereof) and/or due to any between-instrument difference in the weights placed on each dimension. In an attempt to close the gap between a descriptive measure and a preference-based measure, regression-based algorithms discard the equal or ad hoc weighting of descriptive measures and instead weight each item, subscale or scale entering the regression according to the magnitude and direction of association with a preference-based regressand. While the coverage and sensitivity of any two given instruments is unlikely to correspond purely by chance, previous applications of the TTU approach have demonstrated that there is enough commonality between generic descriptive measures and generic preference-based measures to derive a transformation with adequate predictive validity for between-group comparisons [6-10].

For the majority of descriptive condition-specific outcome measures, there is no preference-based alternative with comparable sensitivity and coverage. It is therefore possible that the evidence for generic to generic transformations may not be applicable in the case of condition-specific to generic transformations. Transformation of descriptive condition-specific measures to a generic preference-based measure would typically require mapping from a detailed description of a relatively narrow area of HRQoL space to a general description of the entire HRQoL domain. We might therefore expect a condition-specific to generic transformation to be relatively poor when compared against a generic to generic transformation. However, the validity of this *a priori *expectation is yet to be tested for stroke-specific outcome measures and the extent of any additional error when transforming from descriptive stroke-specific measures to preference-based measures has yet to be quantified.

The purpose of the present study is to demonstrate the feasibility and value of TTU regression in stroke by deriving a transformation from two descriptive stroke-specific measures and a generic measure of health status to a preference-based measure of HRQoL in a sample of Australians with a diagnosis of acute stroke. This will allow quantification of the additional error associated with a condition-specific to generic transformation as compared to a generic to generic transformation in stroke. The resulting transformations will provide a valuable tool for investigators evaluating stroke interventions, potentially widening the set of descriptive stroke-specific measures of HRQoL that can be transformed to preference-based measures for the purposes of economic evaluation.

## Materials and methods

### Data

Data were obtained from the North East Melbourne Stroke Incidence Study (NEMESIS) [11]. The sample for the present study included 926 persons with a diagnosis of acute stroke under the World Health Organization (WHO) definition [12], drawn from a defined area of 22 postcodes in inner northeast Melbourne, Australia during the period May 1, 1996 to April 30, 1999. Further details regarding the study population and case ascertainment are provided elsewhere [11]. The average age of respondents in the study sample was 73.4 years (SD = 13.51), with 51.7% of respondents being female. The NEMESIS study protocol scheduled repeated observations on respondents, with observations available at up to six time points in our 926 respondents. Due to missing data, an AQoL index score paired with a valid scale, subscale or index score on at least one of the SF-36, NIHSS and Barthel could not be derived for all 926 respondents. The 859 participants with a valid AQoL index score for at least one time point paired with a valid scale, subscale or index score on at least one of the SF-36, NIHSS and Barthel for the same time point provided 2570 observations for analysis. Larger or smaller sub-samples were available for the derivation and validation of each algorithm depending on the extent of missing data for the SF-36, NIHSS and Barthel.

### Measures

The preference-based 'target' measure chosen was the Assessment of Quality of Life (AQoL) instrument [13,14] – the only generic preference-based measure of HRQoL that has been scaled and validated in Australia for use in the general population [13,14] and for use in people with stroke [15]. The AQoL descriptive system includes 5 dimensions: illness, independent living, social relationships, physical senses and psychological well-being. Four of the five dimensions and 12 of the 15 items contribute to the preference-based index score, with the illness dimension and associated items excluded because they are indicative of an underlying health condition rather than the impact of that health condition on HRQoL. The AQoL index score varies from -0.04 to 1.00 where unity designates full health, zero designates death, negative scores designate states worse than death, and the lower bound of -0.04 designates the AQoL's 'all worst health state'.

Three descriptive 'base' measures that are commonly used in stroke trials were available for analysis in the present study: the SF-36v1, the National Institutes of Stroke Scale (NIHSS) and the Barthel Index. The SF-36v1 [16,17] is a generic measure of functional health status. It comprises 36 questions in eight subscales or dimensions: Physical Functioning (PF), Role Physical (RP), Bodily Pain (BP), General Health (GH), Vitality (VI), Social Function (SF), Role Emotional (RE) and Mental Health (MH). Each of the eight dimensions is separately scored, using item weighting and additive scaling, to yield a 0–100 point scale. These eight dimensions can be combined into two summary measures – physical function (PCS index) and mental health (MCS index), each on a 0–100 point scale with population means ± standard deviations (SD) equal to 50 ± 10 [17].

The NIHSS [18] measures the severity of physical impairment associated with stroke via a neurological examination across 15 items: level of consciousness (three items), eye movements (one item), visual fields (one item), facial weakness (one item), motor arm strength (two items), motor leg strength (two items), limb ataxia (one item), sensory function (one item), language (one item), articulation (one item), and extinction/inattention (neglect) (one item). Each item is scored from zero (lowest severity) to a maximum of two, three or four (highest severity), and item scores are summed over all items to provide an index of stroke severity that varies from zero (lowest severity) to 42 (highest severity) [18]. The Barthel Index [19] measures disability or functional status based on patient or proxy completion of ten items related to activities of daily living (ADL): feeding, dressing, grooming, bathing, toilet use, transfer, stairs, mobility, bladder, and bowels. Each item is scored from zero (lowest functional status) to a maximum of two), three, or four (highest functional status), and item scores are summed over all items to provide an index of disability on a zero (highest functional status) to 20 (lowest functional status) scale [19].

### Data analysis

We randomly selected approximately 50% of observations available for each algorithm into an estimation set (SF-36 = 1288 observations, NIHSS = 1302 observations, Barthel = 1316 observations), and retained remaining observations in a validation set (SF-36 = 1256 observations, NIHSS = 1268 observations, Barthel = 1252 observations) to allow 'post-sample' but 'within-context' tests of predictive validity. We found no significant difference between estimation and validation sets for SF-36, NIHSS or Barthel datasets with respect to gender (Pearson's chi-square χ^2 ^≤ 0.50, p ≥ 0.48), age (F_SF-36 _= 0.41, p ≥ 0.52; F_NIHSS _= 0.10, p ≥ 0.76; F_Barthel _= 1.57, p ≥ 0.21), health status as measured by the SF-36 MCS (F_SF-36 _= 0.04, p ≥ 0.84), SF-36 PCS (F_SF-36 _= 1.68, p ≥ 0.195), Barthel Index (F_Barthel _= 0.87, p ≥ 0.350), NIHSS (F_NIHSS _= 0.63, p ≥ 0.426), or health-related quality of life as measured by the AQoL (F_SF-36 _= 0.30, p ≥ 0.59; F_NIHSS _= 0.86, p ≥ 0.35; F_Barthel _= 0.73, p ≥ 0.39) where F statistics were obtained from one-way analysis of variance.

We first estimated the relationship between AQoL index scores and the three descriptive measures across the full range of stroke severity using multiple linear regression modelling (the 'all stroke' models). In an attempt to obtain further improvements in predictive validity, we subsequently re-estimated the best of our 'all stroke' models after partitioning the estimation set into NIHSS = 0–6 and NIHSS ≥ 6 subgroups ('severity-specific' models). For item-based algorithms, AQoL utility scores were regressed onto item scores. The inclusion of second-order and interaction terms in the item-based regressions was not practical given degrees of freedom constraints and the large number of first-order terms. In the case of item-based algorithms, we retained first-order terms in the item-based model solely on the basis of their contribution to the regression; as evaluated by the probability of F (enter p ≤ 0.05, remove p ≥ 0.10). For the subscale-, scale- or index-based algorithms, we regressed AQoL utility scores on subscale or scale scores plus interactions and second-order terms in the case of the SF-36, and on index scores plus second-order terms in the case of the NIHSS and Barthel algorithms. For all algorithms, we retained interaction and second-order terms where they made a significant individual or joint contribution to the regression based on the probability of F (enter p ≤ 0.05, remove p ≥ 0.10).

Some previous studies estimating scale- or subscale-based algorithms have retained all first-order terms for reasons of theoretical consistency – irrespective of their individual contributions to the model [9]. We identified some collinearity between SF-36 scale scores in our estimation sample (Pearson's r = 0.085, p < 0.000) but deemed PCS and MCS scores to be sufficiently orthogonal to follow precedent and retain both first-order terms for the scale-based regression. Likewise, index scores for the Barthel and NIHSS algorithms were retained irrespective of their individual contributions to the model. In contrast, the eight SF-36 subscales were highly collinear in the estimation sample such that the omission of one or more subscales from the subscale-based algorithm is consistent with theory. We therefore retained first-order terms in subscale-based regressions solely based on their contribution to the regression as evaluated by the probability of F (enter p ≤ 0.05, remove p ≥ 0.10).

In the survey sample, observations are clustered by respondent such that residuals might be independent between clusters but may not be independent within clusters. The robust Huber/White sandwich estimator is frequently used to adjust for clustering of the residuals in situations where the intra-cluster correlation coefficient is significantly greater than zero. While this approach delivers robust standard errors suitable for calculating confidence intervals, it does not render an inconsistent model (due, for example, to failure to control for respondent-specific effects) consistent [20]. The random effects model explicitly accounts for cluster-specific effects under the assumption that they are independent of other regressors (index, scale, subscale or item scores from the descriptive measure) within the range of the data. The fixed effects error components model controls for respondent specific effects but relaxes the assumption that the cluster-specific effects are uncorrelated with other regressors. A variance partition coefficient: ρ = , can be obtained from the random and fixed effects models to quantify the proportion of residual variance attributable to respondent-specific effects [21]. We used the population-average model where results suggested that respondent-specific effects were quantitatively unimportant. When our results suggested the presence of quantitatively important respondent-specific effects, we chose between fixed and random effects models using Hausman's specification test [[20], p576].

We identify the 'correct' specification within each class of algorithm using standard diagnostic tests. Following Harvey [22], the 'correctness' of each algorithm was evaluated against the criteria of parsimony, identifiability, goodness of fit, theoretical consistency and predictive power. In the present context, theoretical consistency is concerned with (a) obtaining non-negative coefficients on all items, subscales and scales (when coded so that higher item, subscale and scale scores reflect higher levels of HRQoL) and (b) restricting predicted AQoL scores to the -0.04 to 1.0 domain of the target construct. Evaluating the predictive validity of competing algorithms is much more complex than evaluating theoretical consistency but is (minimally) concerned with: (i) strength of association between predicted and observed AQoL scores in the validation sample at the individual-level, (ii) deviation between predicted and observed AQoL scores at the individual level in the validation sample, (iii) deviation between predicted and observed AQoL scores at the group level in the validation sample.

With regards to (i), the higher the strength of association, the better the algorithm is able to predict variation along the scale. Note, however, that "two measures can be perfectly correlated but have poor agreement" [[23], p977]. We might be relatively confident that a high score on the predicted AQoL scale would be mirrored by a high score on the observed AQoL scale but there is no guarantee that the two scales are compressed between the same limits. With regards to (ii), a summary measure of the deviation between predicted and observed scores at the individual level such as the mean absolute difference (MAD) indicates the average precision with which we can predict an individual's AQoL score. We calculated MADs by taking the absolute difference between predicted and observed scores for each individual, summing over all individuals, and dividing through by the total number of observations.

While a high degree of precision in predicting AQoL scores at the individual level would imply a high level of precision with respect to other criteria, such precision might not be necessary for the sort of between-group comparisons that form the basis for estimates of both treatment effects and health-state utilities. Specifically, errors at the individual level might not translate into errors at the group level such that minimising the deviation between predicted and observed AQoL utility scores *at the group level *is all that is required. For the purposes of evaluating precision at the group level in the present study, we split the study sample into three sub-groups defined by stroke severity on the NIHSS (0; 1–5; and ≥ 6). While (iii) is the most relevant test of predictive validity in measuring group-level treatment effects and health-state utilities, we report findings on all three criteria to provide a more complete evaluation of the strengths and weaknesses of our transformations. We conducted the analyses reported here using SPSS 15.0 for Windows [24] and STATA/SE 8.2 for Windows [25].

## Results

Table 1 describes the demographic characteristics for observations (rather than respondents) and the distribution of AQoL, NIHSS, SF-36 and Barthel scores for the study sample used to derive and validate each algorithm. The mean AQoL score across all observations was 0.47 (SD = 0.34), demonstrating the vastly poorer health-related quality of life of people with stroke as compared with the population norm of 0.83 in the Australian non-institutionalised population [13]. Model fit, estimated coefficients and post-sample tests of predictive validity are summarised below for 'all stroke' and 'severity-specific' algorithms.

**Table 1:** Descriptive statistics on observations

	N(%)	Min	Max	Mean	SD
**SF-36 to AQoL algorithm**					

Female	1257(49)	-	-	-	-

Age	2543	2.26	98.13	71.528	13.511

AQoL					

Utility Score	2544	-0.04	1.00	0.467	0.338

SF-36 Scales					

PCS	2119	4.46	68.38	38.040	11.724
MCS	2119	5.57	75.49	49.614	11.941

SF-36 Subscales					

Physical Function (PF)	2132	0	100	44.308	34.731
Role Physical (RP)	2132	0	100	51.466	44.552
Bodily Pain (BP)	2132	0	100	74.546	27.671
General Health (GH)	2126	0	100	56.247	25.141
Vitality (VI)	2128	0	100	49.039	24.113
Social Function (SF)	2132	0	100	71.582	34.010
Role Emotional (RE)	2127	0	100	76.399	39.766
Mental Health (MH)	2128	0	100	73.085	21.383

**Barthel to AQoL algorithm**					

Female	1242(48)	-	-	-	-

Age	2510	2.26	98.13	71.520	13.522

AQoL					

Utility Score	2568	-0.04	1.00	0.467	0.338

Barthel Index					

Barthel Index Score	2568	0	20	15.859	6.191

**NIHSS to AQoL algorithm**					

Female	1275(49)	-	-	-	-

Age	2570	2.26	98.13	71.613	13.481

AQoL					

Utility Score	2570	-0.04	1.00	0.467	0.338

NIHSS					

NIHSS Total	2561	0	29	1.595	3.564

### Conversion of SF-36 scale scores to QALY-weights

Table 2 summarises parameter estimates and model fit for the fixed effects, scale-based SF36 algorithm. The intra-cluster correlation coefficient for AQoL scores in the estimation sample (ICC = 0.733, 95%CI: 0.69, 0.77) suggested that some adjustment should be made for clustering by individual. Results from the fixed effects error components model confirm that a significant proportion of variation is attributable to respondent-specific effects (ρ = 0.706) and that respondent-specific fixed effects are significantly greater than zero (F = 2.85, df = (639,431), p < 0.000) [21]. The Hausman specification test for the appropriateness of the random effects estimator rejected the null hypothesis of no systematic differences between coefficients from fixed and random effects models (χ^2 ^= 68.77, df = 3, p < 0.000), implying that the additional assumptions required by the random effects model were not met in the estimation sample.

**Table 2:** Regression algorithms for converting SF-36 scores into AQoL scores

**Model**	**Predictor**	**β**	**SE**	**t**	**Sig.**
SF-36 Scale					

All stroke	(Constant)	0.1148	0.139	0.82	0.411
	PCS	0.0024	0.003	0.67	0.503
	MCS	-0.0004	0.003	-0.14	0.885
	PCS*PCS				ns
	MCS*MCS				ns
	MCS*PCS	0.0001	0.000	2.23	0.027
	
		0.7056		F_639,431 _= 2.85	0.000
	
		Obs^ = 1074	Ids^# ^= 640	F_3,431 _= 37.01	0.000
	
			R^2^_within _= 0.21	R^2^_between _= 0.59	R^2^_overall _= 0.55

SF-36 Subscale					

All stroke	(Constant)	0.0986	0.314	3.15	0.002
	Physical Function (PF)	0.0057	0.001	4.46	0.000
	General Health (GH)	0.0017	0.001	3.11	0.002
	Mental Health (MH)*PF	3.84*10^-5^	9.73*10^-6^	3.95	0.000
	PF*PF	-5.35*10^-5^	1.19*10^-5^	-4.48	0.000
	PF* Role Physical (RP)	1.29*10^-5^	6.11*10^-6^	2.11	0.035
	Social Function (SF)*SF	8.47*10^-6^	2.56*10^-6^	3.31	0.001
	Bodily Pain (BP)*RP	8.65*10^-6^	4.35*10^-6^	1.99	0.047
	GH*RP	-2.10*10^-5^	6.35*10^-6^	-3.30	0.001
	
		0.6298		F_639,431 _= 2.01	0.000
	
		Obs = 1079	Ids = 640	F_8,431 _= 28.78	0.000
	
			R^2^_within _= 0.35	R^2^_between _= 0.75	R^2^_overall _= 0.72

SF-36 Item					

All stroke	(Constant)	-0.1986	0.0790	-2.51	0.012
	Item 1 (general health now)	-0.0197	0.0101	-1.94	0.053
	Item 3b (moderate activities)	0.0519	0.0151	3.44	0.001
	Item 3e (one flight stairs)	0.0353	0.0160	2.21	0.028
	Item 3h (walking 1/2 km)	0.0345	0.0155	2.22	0.027
	Item 3j (bathing/dressing)	0.0768	0.0173	4.43	0.000
	Item 4a (other activities)	0.0279	0.0168	1.67	0.096
	Item 9b (nervous)	0.0157	0.0066	2.37	0.018
	Item 9f (felt down)	0.0132	0.0075	1.74	0.082
	Item 9i (tired)	0.0199	0.0065	3.04	0.002
	Item 10 (social activities, time)	0.0147	0.0064	2.31	0.021
	
		0.6294		F_640,429 _= 1.85	0.000
	
		Obs = 1080	Ids = 641	F_10,429 _= 21.87	0.000
	
			R^2^_within _= 0.34	R^2^_between _= 0.73	R^2^_overall _= 0.71

^Obs denotes number of observations. ^#^Ids denotes number of respondents.

Post-sample tests of predictive validity for fixed effects, scale-based SF36 to AQoL algorithm are reported in Table 3. Mean predicted AQoL utility scores were not significantly different from their corresponding mean observed scores in all stroke (t = 0.000, p = 1.000) patients or for the NIHSS = 1–5 (t = -0.572, p = 0.567) subgroup but the presence of significant differences in NIHSS = 0 (t = 2.662, p = 0.0079) and NIHSS ≥ 6 subgroups (t = -11.704, p = 0.000) suggests that averaging over all groups masks errors at the group level. The predictive validity of the scale-based algorithm was therefore deemed inadequate for the sort of between-group comparisons required for evaluating the effectiveness and cost-effectiveness of interventions.

**Table 3:** Post-sample predictive validity for 'all stroke' SF-36 to AQoL algorithms

Data	Model	Group	N	Min	Max	Mean	SD
Observed AQoL	Validation sample	NIHSS = 0	786	-0.04	1.00	0.529	0.334
		NIHSS = 1–5	337	-0.04	1.00	0.440	0.296
		NIHSS ≥ 6	114	-0.04	1.00	0.112	0.205
		Missing	19	-0.03	1.00	0.278	0.357
		Total	1256	-0.04	1.00	0.464	0.337

Predicted AQoL	Scale-based	NIHSS = 0	580	0.20	0.75	0.494	0.134
		NIHSS = 1–5	334	0.21	0.73	0.450	0.123
		NIHSS ≥ 6	112	0.22	0.66	0.361	0.097
		Missing	19	0.25	0.73	0.403	0.141
		Total	1045	0.20	0.75	0.464	0.134
	Subscale-based	NIHSS = 0	580	0.10	0.79	0.523	0.193
	
		NIHSS = 1–5	334	0.12	0.80	0.456	0.185
		NIHSS ≥ 6	112	0.10	0.73	0.262	0.144
		Missing	19	0.10	0.73	0.346	0.206
		Total	1045	0.10	0.80	0.460	0.202
	
	Item-based	NIHSS = 0	581	0.05	0.80	0.513	0.191
		NIHSS = 1–5	335	-0.01	0.78	0.453	0.185
		NIHSS ≥ 6	112	0.02	0.72	0.262	0.150
		Missing	19	0.11	0.77	0.363	0.215
		Total	1047	-0.01	0.80	0.464	0.200

Mean Absolute Deviation (MAD)	Scale-based	NIHSS = 0	580	0.00	0.54	0.215	0.120
		NIHSS = 1–5	334	0.00	0.62	0.196	0.123
		NIHSS ≥ 6	112	0.01	0.49	0.280	0.097
		Missing	19	0.03	0.45	0.246	0.132
		Total	1045	0.00	0.62	0.216	0.121
	
	Subscale-based	NIHSS = 0	580	0.00	0.77	0.164	0.109
		NIHSS = 1–5	334	0.00	0.62	0.161	0.117
		NIHSS ≥ 6	112	0.01	0.56	0.184	0.103
		Missing	19	0.04	0.33	0.176	0.080
		Total	1045	0.00	0.77	0.165	0.111
	
	Item-based	NIHSS = 0	581	0.00	0.65	0.163	0.109
		NIHSS = 1–5	335	0.00	0.68	0.181	0.117
		NIHSS ≥ 6	112	0.01	0.68	0.181	0.117
		Missing	19	0.03	0.36	0.175	0.102
		Total	1047	0.00	0.68	0.163	0.111

There is also only a weak correspondence between predicted and observed scores at the individual level. For example, a high proportion (79.4%) of absolute deviations between predicted and observed scores were in excess of 0.10 on the AQoL scale. Likewise, correlations between predicted and observed AQoL utility scores in the validation sample for all stroke (Pearson's r = 0.750), NIHSS = 0 (Pearson's r = 0.744), NIHSS = 1–5 (Pearson's r = 0.676), and NIHSS ≥ 6 groups (Pearson's r = 0.635) were on par with those reported for existing conversion algorithms but are not sufficiently strong to imply that predicted AQoL scores provide an adequate proxy for directly observed AQoL scores at the individual level [9].

### Conversion of SF-36 subscale scores to QALY-weights

Parameter estimates and model fit for the subscale-based SF36 algorithm are reported in Table 2. Respondent-specific fixed effects were again significantly greater than zero (F = 2.01, df = (639,431), p < 0.000) and the Hausman specification test (χ^2 ^= 39.87, df = 8, p < 0.000) again suggested that the fixed effects model most appropriately characterised respondent-specific effects. Post-sample tests of predictive validity for the subscale-based SF36 to AQoL algorithm are reported in Table 3. Mean predicted AQOL utility scores were not significantly different from their corresponding mean observed scores in all stroke (t = 0.352, p = 0.725) patients or in the NIHSS = 0 (t = 0.418, p = 0.676) and NIHSS = 1–5 (t = -0.840, p = 0.401) subgroups. However, a significant difference between observed and predicted AQoL scores in the NIHSS ≥ 6 subgroup (t = -6.374, p < 0.000) implies that the predictive validity of the subscale-based algorithm was inadequate for between-group comparisons across the full range of stroke severity.

Partitioning the sample and running separate regressions for the NIHSS = 0–5 ('low severity') and NIHSS ≥ 6 ('moderate to high severity') subgroups produced an improvement in model fit and predictive validity. Table 4 summarises model fit and estimated coefficients for 'low severity' and 'moderate to high severity' subscale-based conversion algorithms. Table 5 summarises post-sample tests of predictive validity for these 'severity-specific' subscale-based conversion algorithms. For the 'low severity' algorithm, respondent-specific fixed effects were significantly greater than zero (F = 2.14, df = (566,364), p < 0.000) and the Hausman specification test (χ^2 ^= 33.92, df = 10, p < 0.000) suggested that the fixed effects model most appropriately characterised respondent-specific effects. Results from random and fixed effects models (not reported here) for the 'moderate to high severity' algorithm suggest that the proportion of variance attributable to respondent specific effects is approximately zero. Model fit and estimated coefficients for the 'moderate to high severity' algorithm are therefore drawn from the population-average model.

**Table 4:** Severity-specific algorithms for converting SF-36 data into AQoL scores

**Model**	**Predictor**	**β**	**SE**	**t**	**Sig.**
SF-36 Subscale					

NIHSS = 0–5	(Constant)	0.0364	0.0423	0.86	0.390
	Physical Function (PF)	0.0074	0.0014	5.24	0.000
	Bodily Pain (BP)	0.0006	0.0004	1.81	0.072
	Social Function (SF)	0.0022	0.0007	3.12	0.002
	PF*PF	-5.25*10^-5^	1.22*10^-5^	-4.29	0.000
	PF*Mental Health (MH)	2.90*10^-5^	1.36*10^-5^	2.13	0.034
	Vitality (VI)*VI	-1.69*10^-5^	7.20*10^-6^	-2.35	0.019
	VI*Role Physical (RP)	3.79*10^-5^	9.47*10^-6^	4.00	0.000
	General Health (GH)*MH	2.49*10^-5^	8.61*10^-6^	2.89	0.004
	GH*RP	-3.07*10^-5^	7.89*10^-6^	-3.89	0.000
	SF*MH	-1.61*10^-5^	9.71*10^-6^	-1.66	0.097
	
		0.6346		F_566,364 _= 2.14	0.000
	
		Obs = 941	Ids = 567	F_10,364 _= 22.34	0.000
	
			R^2^_within _= 0.38	R^2^_between _= 0.69	R^2^_overall _= 0.67

NIHSS ≥ 6	(Constant)	0.0744	0.0781	0.95	0.343
	BP*SF	-2.23*10^-5^	7.60*10^-6^	-2.93	0.004
	PF	0.0081	0.0023	3.52	0.001
	RP	-0.0030	0.0013	-2.29	0.024
	MH*MH	-2.80*10^-5^	1.29*10^-5^	-2.17	0.032
	VI	-0.0053	0.0031	-1.68	0.096
	SF*PF	7.89*10^-5^	2.41*10^-5^	3.27	0.002
	PF*PF	-0.0001	1.49*10^-5^	-8.38	0.000
	PF*RP	-7.79*10^-5^	1.86*10^-5^	-4.20	0.000
	MH*RP	6.49*10^-5^	2.82*10^-5^	2.30	0.000
	SF*SF	1.84*10^-5^	6.65*10^-6^	2.77	0.007
	VI*MH	8.90*10^-5^	4.27*10^-5^	2.09	0.040
	GH*MH	1.85*10^-5^	9.26*10^-6^	1.99	0.049
	
		-	-	-	ns
	
		Obs = 117	Ids = 96	F_12,95 _= 35.12	0.000
	
					R^2^_overall _= 0.50

SF-36 Item					

NIHSS = 0–5	(Constant)	-0.2424	0.0757	-3.20	0.001
	Item 2 (general health change)	-0.0408	0.0153	-2.67	0.008
	Item 3b (moderate activities)	0.0584	0.0156	3.74	0.000
	Item 3d (several flights stairs)	0.0321	0.0154	2.09	0.038
	Item 3h (walking 1/2 km)	0.0384	0.0159	2.42	0.016
	Item 3j (bathing/dressing)	0.0934	0.0175	5.35	0.000
	Item 4a (other activities)	0.0590	0.0215	2.74	0.006
	Item 4b (accomplished less)	-0.0386	0.0220	-1.75	0.080
	Item 9b (nervous)	0.0195	0.0072	2.70	0.007
	Item 9f (felt down)	0.0159	0.0085	1.88	0.061
	Item 9i (tired)	0.0250	0.0069	3.60	0.000
	Item 10 (social activities, time)	0.0224	0.0068	-3.20	0.001
	
		0.6378		F_567,363 _= 2.05	0.000
	
		Obs = 942	Ids = 568	F_11,363 _= 20.68	0.000
	
			R^2^_within _= 0.39	R^2^_between _= 0.69	R^2^_overall _= 0.67

NIHSS ≥ 6	(Constant)	0.0331	0.0427	0.77	0.441
	Item 3a (vigorous activities)	-0.1897	0.0497	-3.82	0.000
	Item 3b (moderate activities)	-0.2940	0.1393	-2.11	0.037
	Item 3d (several flights stairs)	0.1462	0.0623	2.35	0.021
	Item 3g (walking > 1 km)	0.2080	0.0828	2.51	0.014
	Item 3j (bathing/dressing)	0.0901	0.0251	3.58	0.001
	Item 6 (social activities, extent)	-0.0139	0.0082	-1.69	0.094
	Item 9c (down in dumps)	0.0135	0.0068	1.99	0.050
	Item 11c (expect worse health)	0.0163	0.0066	2.46	0.016
	
		-	-	-	ns
	
		Obs = 117	Ids = 96	F_8,95 _= 15.44	0.000
	
					R^2^_overall _= 0.37

**Table 5:** Post-sample predictive validity for 'severity-specific' SF-36 to AQoL algorithms

Data	Model	Group	N	Min	Max	Mean	SD
Observed AQoL	Validation sample	NIHSS = 0	786	-0.04	1.00	0.529	0.334
		NIHSS = 1–5	337	-0.04	1.00	0.440	0.296
		NIHSS ≥ 6	114	-0.04	1.00	0.112	0.205

Predicted AQoL	Subscale-based	NIHSS = 0*	580	-0.05	0.93	0.523	0.266
		NIHSS = 1–5*	334	-0.02	0.92	0.450	0.252
		NIHSS ≥ 6^	112	-1.17	0.68	0.105	0.205
	
	Item-based	NIHSS = 0*	581	-0.08	0.90	0.532	0.264
		NIHSS = 1–5*	335	-0.16	0.93	0.447	0.261
		NIHSS ≥ 6^	112	-0.21	0.72	0.114	0.150

Mean Absolute Deviation (MAD)	Subscale-based	NIHSS = 0*	580	0.00	0.76	0.137	0.115
		NIHSS = 1–5*	334	0.00	0.73	0.149	0.122
		NIHSS ≥ 6^	112	0.00	1.14	0.125	0.179
	
	Item-based	NIHSS = 0*	581	0.00	0.78	0.130	0.111
		NIHSS = 1–5*	335	0.00	0.76	0.141	0.114
		NIHSS ≥ 6^	112	0.00	0.74	0.095	0.122

*Predicted values obtained from 'low severity' algorithm. ^Predicted values obtained from 'moderate to severe severity' algorithm

Mean predicted AQoL utility scores were not significantly different from their corresponding mean observed scores in NIHSS = 0 (t = 0.357, p = 0.721), NIHSS = 1–5 (t = -0.471, p = 0.638) and NIHSS ≥ 6 (t = -0.257, p = 0.798) subgroups when the 'low severity' algorithm is used to predict AQoL scores for patients in the NIHSS = 0 and NIHSS = 1–5 subgroups, and the 'moderate to severe severity' algorithm is used to predict AQoL scores for patients in the NIHSS ≥ 6 subgroup. For all subgroups, the difference between mean predicted and mean observed scores was less than 0.01 on the AQoL scale – a magnitude of error that is unlikely to mask minimally important differences (MIDs) for between-group or pre-post treatment effects [26]. While the predictive validity of the item-based SF-36 to AQoL algorithm is now adequate for between-group comparisons, the mean absolute deviations reported in Table 5 imply that the subscale-based algorithm is not sufficiently precise for the purposes of predicting health state utilities or change scores at the individual level.

### Conversion of SF-36 item scores to QALY-weights

Parameter estimates and model fit for the fixed effects, item-based SF36 to AQoL algorithm are reported in Table 2. Respondent-specific fixed effects were again significantly greater than zero (F = 1.85, df = (640,429), p < 0.000) and the Hausman test (χ^2 ^= 55.32, df = 10, p < 0.000) again suggested that the fixed effects model most appropriately characterised respondent-specific effects. Post-sample tests of predictive validity are reported in Table 3. Mean predicted AQoL utility scores were not significantly different at the 0.05 level from their corresponding mean observed scores in all stroke (t = 0.000, p = 1.000) patients or in the NIHSS = 0 (t = 1.036, p = 0.300) and NIHSS = 1–5 (t = -0.682, p = 0.495) subgroups. However, a significant difference between observed and predicted AQoL scores in the NIHSS ≥ 6 subgroup (t = -6.269, p < 0.000) suggests that the predictive validity of the subscale-based algorithm was inadequate for patients at the more severe end of the scale.

Partitioning the sample and running separate regressions for the NIHSS = 0–5 ('low severity') and NIHSS ≥ 6 ('moderate to high severity') subgroups produced an improvement in predictive validity. Results from random and fixed effects models (not reported here) for the 'moderate to high severity' algorithm suggest that the proportion of variance attributable to respondent specific effects is approximately zero. Model fit and estimated coefficients for the 'moderate to high severity' algorithm derived in the NIHSS ≥ 6 subgroup and reported in Table 4 are therefore drawn from a group-average estimator. Table 5 summarises post-sample tests of predictive validity for 'severity-specific', item-based conversion algorithms. For the 'low severity' algorithm, respondent-specific fixed effects were significantly greater than zero (F = 2.05, df = (567,363), p < 0.000) and the Hausman test (χ^2 ^= 46.64, df = 11, p < 0.000) suggested that the fixed effects model most appropriately characterised respondent-specific effects.

Comparison between mean predicted and mean observed AQoL utility scores by subgroup now suggests that the predictive validity of the item-based SF-36 algorithms is adequate for between-group comparisons when the 'low severity' algorithm is used to predict AQoL scores for patients in the NIHSS = 0 and NIHSS = 1–5 subgroups and the 'moderate to severe severity' algorithm is used to predict AQoL scores for patients in the NIHSS ≥ 6 subgroup. Mean predicted AQoL utility scores were not significantly different from their corresponding mean observed scores in NIHSS = 0 (t = -0.185, p = 0.853), NIHSS = 1–5 (t = -0.325, p = 0.745) and NIHSS ≥ 6 (t = -0.084, p = 0.933) subgroups. The difference between mean predicted and mean observed scores was less than 0.01 on the AQoL scale for all subgroups – a magnitude of error that is unlikely to mask minimally important differences (MIDs) for between-group or pre-post treatment effects [26]. While the predictive validity of the item-based SF-36 to AQoL algorithm is now adequate for between-group comparisons, MADs in excess of 0.10 for NIHSS = 0 and NIHSS = 1–5 subgroups imply that partitioning the sample fails to remedy errors at the individual level. Item-based SF-36 algorithms therefore remain insufficiently precise for the purposes of predicting health state utilities or change scores for individual patients.

### Conversion of NIHSS index and item scores to QALY-weights

The index-based NIHSS algorithm failed to reach statistical significance at the 0.05 level in the full study sample (F = 1.35, df = (2,595), p = 0.259). Partitioning the sample and running separate regressions for the NIHSS = 0–5 ('low severity') and NIHSS ≥ 6 ('moderate to high severity') subgroups produced an improvement in model fit and predictive validity for index-based NIHSS algorithms. Parameter estimates and model fit for the index-based NIHSS 'all stroke' and 'severity-specific' algorithms are given in Table 6. The Hausman test suggested that the fixed effects model most appropriately characterised respondent-specific effects in the NIHSS = 0 and NIHSS = 1–5 (χ^2 ^= 49.53, df = 2, p < 0.000) subgroups whereas the additional assumptions required for the random effects model were met in the NIHSS ≥ 6 subgroup (χ^2 ^= 0.83, df = 2, p = 0.660).

**Table 6:** Regression algorithms for converting NIHSS data into AQoL scores

**Model**	**Predictor**	**β**	**SE**	**t**	**Sig.**
NIHSS Index					

All stroke	(Constant)	0.4639	0.0044	104.97	0.000
	NIHSS	0.0024	0.0020	1.20	0.230
	NIHSS*NIHSS	-0.0000	0.0000	-1.22	0.224
	
		0.7856		F_849,1718 _= 8.45	0.000
	
		Obs = 1302	Ids = 705	F_2,595 _= 1.35	0.259
	
			R^2^_within _= 0.00	R^2^_between _= 0.17	R^2^_overall _= 0.12

NIHSS = 0–5	(Constant)	0.4754	0.0066	72.07	0.000
	NIHSS	0.0802	0.0178	4.52	0.000
	NIHSS*NIHSS	-0.0170	0.0046	-3.68	0.000
	
		0.7955		F_652,540 _= 6.27	0.000
	
		Obs = 1195	Ids = 653	F_2,540 _= 11.41	0.000
	
			R^2^_within _= 0.04	R^2^_between _= 0.00	R^2^_overall _= 0.00

NIHSS ≥ 6	(Constant)	0.2882	0.0874	3.30	0.001
	NIHSS	-0.0247	0.0133	-1.85	0.064
	NIHSS*NIHSS	0.0005	0.0004	1.19	0.234
	
		0.7680	-	-	-
	
		Obs = 103	Ids = 88	Wald χ^2 ^= 11.58	0.003
	
			R^2^_within _= 0.00	R^2^_between _= 0.12	R^2^_overall _= 0.12

NIHSS Item					

All stroke	(Constant)	0.4499	0.0059	76.81	0.000
	Visual fields	-0.0475	0.0232	-2.05	4.53
	Facial weakness	0.0909	0.0201	4.53	0.000
	
		0.8103		F_704,595 _= 6.86	0.000
	
		Obs = 1302	Ids = 705	F_2,595 _= 10.89	0.000
	
			R^2^_within _= 0.04	R^2^_between _= 0.03	R^2^_overall _= 0.01

NIHSS = 0–5	(Constant)	0.4810	0.0055	88.15	0.000
	Facial weakness	0.0984	0.0232	4.24	0.000
	Limb ataxia	0.0630	0.0273	2.31	0.021
	
		0.7984		F_652,540 _= 6.67	0.000
	
		Obs = 1195	Ids = 653	F_2,540 _= 12.42	0.000
	
			R^2^_within _= 0.04	R^2^_between _= 0.01	R^2^_overall _= 0.01

NIHSS ≥ 6	(Constant)	0.0732	0.0496	1.48	0.191
	Consciousness	-0.3052	0.0572	-5.34	0.002
	Eye movements	0.3073	0.0846	3.63	0.011
	Facial weakness	-0.1033	0.0263	-3.93	0.008
	Motor – Left arm	0.0760	0.0208	3.65	0.011
	Motor – Right leg	-0.3157	0.0364	-8.66	0.000
	Motor – Left leg	0.2980	0.0415	7.18	0.000
	Sensory	-0.1340	0.0310	-4.33	0.005
	Language	0.1336	0.0319	4.19	0.006
	Extinction/Inattention	-0.1198	0.0270	-4.44	0.004
	
		0.6653		F_87,6 _= 32.07	0.000
	
		Obs = 103	Ids = 88	F_9,6 _= 10.36	0.005
	
			R^2^_within _= 0.94	R^2^_between _= 0.05	R^2^_overall _= 0.05

For the item-based NIHSS algorithms, the Hausman test suggested that the fixed effects model most appropriately characterised respondent-specific effects for the all stroke (χ^2 ^= 40.24, df = 2, p < 0.000), NIHSS = 0–5 (χ^2 ^= 23.82, df = 2, p < 0.000) and NIHSS ≥ 6 (χ^2 ^= 76.61, df = 9, p = 0.000) algorithms. With the exception of predictions for the NIHSS ≥ 6 subgroup from the 'moderate to high severity' algorithm, mean predicted AQoL utility scores from item- and index-based NIHSS algorithms were always significantly different from their corresponding mean observed scores. For example, predicted and observed AQoL scores from the index-based NIHSS algorithm were significantly different from one another for NIHSS = 0 (t = 6.084, p = 0.000) and NIHSS = 1–5 (t = -5.732, p = 0.000) but not for the NIHSS ≥ 6 (t = 1.018, p = 0.309) groups. None of the NIHSS-based algorithms can therefore be said to predict AQoL group means with sufficient precision for the purposes of evaluating the effectiveness and cost-effectiveness of interventions. Moreover, MADs for the NIHSS algorithms reported in Table 7 are never lower than 0.120 and as high as 0.307 for some subgroups, nearly one third of the AQoL scale and considerably higher than the mean absolute deviations for the subscale- and item-based SF-36 algorithms reported in Tables 3 and 5. These results suggest that the NIHSS algorithms derived in the present study yielded predicted AQoL scores with such poor correspondence to observed scores that they should not be used for any purpose.

**Table 7:** Post-sample predictive validity for NIHSS 'all stroke' & 'severity-specific' algorithms

Data	Model	Group	N	Min	Max	Mean	SD
Observed AQoL	Validation sample	NIHSS = 0	819	-0.04	1.00	0.546	0.334
		NIHSS = 1–5	312	-0.03	1.00	0.443	0.294
		NIHSS ≥ 6	132	-0.04	0.98	0.112	0.210

All stroke algorithm							

Predicted AQoL	Index-based	NIHSS = 0	819	0.45	0.45	0.453	0.000
		NIHSS = 1–5	312	0.46	0.48	0.466	0.007
		NIHSS ≥ 6	132	0.49	0.57	0.504	0.020
	
	Item-based	NIHSS = 0	819	0.44	0.44	0.443	0.000
		NIHSS = 1–5	312	0.22	0.47	0.435	0.042
		NIHSS ≥ 6	132	0.22	0.47	0.428	0.061

Mean Absolute Deviation (MAD)	Index-based	NIHSS = 0	819	0.00	0.55	0.309	0.156
		NIHSS = 1–5	312	0.00	0.54	0.258	0.147
		NIHSS ≥ 6	132	0.02	0.60	0.431	0.124
	
	Item-based	NIHSS = 0	819	0.00	0.56	0.312	0.157
		NIHSS = 1–5	312	0.00	0.65	0.251	0.148
		NIHSS ≥ 6	132	0.04	0.65	0.114	0.359

Severity algorithms							

Predicted AQoL	Index-based	NIHSS = 0*	819	0.48	0.48	0.475	0.000
		NIHSS = 1–5*	312	0.45	0.57	0.539	0.033
		NIHSS ≥ 6^	132	-0.02	0.16	0.099	0.054
	
	Item-based	NIHSS = 0*	819	0.46	0.46	0.461	0.000
		NIHSS = 1–5*	312	0.46	0.65	0.486	0.032
		NIHSS ≥ 6^	132	-0.08	0.20	0.096	0.046

Mean Absolute Deviation (MAD)	Index-based	NIHSS = 0*	819	0.00	0.52	0.304	0.155
		NIHSS = 1–5*	312	0.00	0.58	0.262	0.160
		NIHSS ≥ 6^	132	0.00	0.82	0.120	0.157
	
	Item-based	NIHSS = 0*	819	0.00	0.54	0.307	0.155
		NIHSS = 1–5*	312	0.00	0.55	0.259	0.146
		NIHSS ≥ 6^	132	0.00	0.65	0.302	0.154

*Predicted values obtained from 'low severity' algorithm. ^Predicted values obtained from 'moderate to severe severity' algorithm.

### Conversion of Barthel index and item scores to QALY-weights

Parameter estimates and model fit for the index- and item-based 'all stroke' Barthel algorithms are given in Table 8. Post-sample tests of predictive validity for the index- and item-based 'all stroke' Barthel algorithms are reported in Table 9. Neither the index- nor item-based 'all stroke' Barthel algorithms provided sufficient predictive power for the purposes of economic evaluation. Mean predicted AQoL utility scores from both item- and index-based 'all stroke' Barthel algorithms were always significantly different from their corresponding mean observed scores in at least one subgroup. Specifically, predicted and observed AQoL scores were significantly different for index-based (t ≥ 3.063, p ≤ 0.002) and item-based (t ≥ 3.056, p ≤ 0.002) 'all stroke' Barthel algorithms in the NIHSS = 0 and NIHSS ≥ 6 subgroups.

**Table 8:** Regression algorithms for converting Barthel data to AQoL scores

**Model**	**Predictor**	**β**	**SE**	**t**	**Sig.**
Barthel Index					

All stroke	(Constant)	0.1817	0.0393	4.63	0.000
	Barthel	-0.0180	0.0070	-2.56	0.011
	Barthel*Barthel	0.0020	0.0003	6.38	0.000
	
		0.6536		F_652,597 _= 2.66	0.000
	
		Obs = 1252	Ids = 653	F_2,597 _= 80.00	0.000
	
			R^2^_within _= 0.211	R^2^_between _= 0.689	R^2^_overall _= 0.631

NIHSS = 0–5	(Constant)	0.2068	0.0471	4.39	0.000
	Barthel	-0.0201	0.0081	-2.47	0.014
	Barthel*Barthel	0.0020	0.0003	4.39	0.000
	
		0.6579		F_597,528 _= 2.75	0.000
	
		Obs = 1128	Ids = 598	F_2,528 _= 67.43	0.000
	
			R^2^_within _= 0.203	R^2^_between _= 0.639	R^2^_overall _= 0.581

NIHSS ≥ 6	(Constant)	0.0071	0.0089	0.80	0.425
	Barthel	-0.0053	0.0067	-0.80	0.429
	Barthel*Barthel	0.0017	0.0004	3.81	0.000
	
		-	-	-	ns
	
		Obs = 120	Ids = 96	F_2,95 _= 51.27	0.000
	
					R^2^_overall _= 0.574

Barthel Item					

All stroke	(Constant)	0.1160	0.0335	3.47	0.001
	Feeding	0.0450	0.0192	2.35	0.019
	Dressing	0.0631	0.0168	3.76	0.000
	Bathing	0.1173	0.0280	4.18	0.000
	Stairs	0.0520	0.0119	4.35	0.000
	Bladder	0.0249	0.0135	1.85	0.065
	
		0.6467		F_652,594 _= 2.54	0.000
	
		Obs = 1252	Ids = 653	F_5,594 _= 30.48	0.000
	
			R^2^_within _= 0.204	R^2^_between _= 0.693	R^2^_overall _= 0.631

NIHSS = 0–5	(Constant)	0.1273	0.0411	3.10	0.002
	Feeding	0.0460	0.0230	2.00	0.046
	Dressing	0.0620	0.0184	3.36	0.001
	Bathing	0.1087	0.0302	3.60	0.000
	Stairs	0.0531	0.0128	4.15	0.000
	Bladder	0.0291	0.0151	1.93	0.054
	
		0.6534		F_597,525 _= 2.66	0.000
	
		Obs = 1128	Ids = 598	F_5,525 _= 25.64	0.000
	
			R^2^_within _= 0.196	R^2^_between _= 0.644	R^2^_overall _= 0.579

NIHSS ≥ 6	(Constant)	-0.0114	0.0103	-1.11	0.269
	Feeding	0.0341	0.0124	2.74	0.007
	Bathing	0.3176	0.0612	5.19	0.000
	Transfer	0.0368	0.0150	2.45	0.016
	Stairs	0.0553	0.0278	1.99	0.049
	
		-	-	-	ns
	
		Obs = 120	Ids = 96	F_4,95 _= 38.02	0.000
	
					R^2^_overall _= 0.639

**Table 9:** Post-sample predictive validity for Barthel 'all stroke' algorithms

Model	Criteria	Group	N	Min	Max	Mean	SD
Observed AQoL	Validation sample	NIHSS = 0	844	-0.04	1.00	0.536	0.334
		NIHSS = 1–5	352	-0.04	1.00	0.446	0.299
		NIHSS ≥ 6	113	-0.04	0.98	0.111	0.199
		Missing	7	-0.03	0.10	0.023	0.053
		Total	1316	-0.04	1.00	0.473	0.337

Predicted AQoL	Index-based	NIHSS = 0	844	0.14	0.61	0.497	0.159
		NIHSS = 1–5	352	0.14	0.61	0.480	0.155
		NIHSS ≥ 6	113	0.14	0.61	0.236	0.128
		Missing	7	0.14	0.31	0.179	0.062
		Total	1316	0.14	0.61	0.469	0.173
	
	Item-based	NIHSS = 0	844	0.12	0.60	0.497	0.161
		NIHSS = 1–5	352	0.12	0.60	0.479	0.155
		NIHSS ≥ 6	113	0.12	0.60	0.231	0.138
		Missing	7	0.12	0.26	0.202	0.046
		Total	1316	0.12	0.60	0.467	0.174

Mean Absolute Deviation (MAD)	Index-based	NIHSS = 0	844	0.00	0.59	0.198	0.118
		NIHSS = 1–5	352	0.00	0.62	0.191	0.132
		NIHSS ≥ 6	113	0.00	0.77	0.170	0.109
		Missing	7	0.04	0.32	0.156	0.097
		Total	1316	0.00	0.77	0.193	0.121
	
	Item-based	NIHSS = 0	844	0.00	0.59	0.196	0.119
		NIHSS = 1–5	352	0.00	0.59	0.189	0.130
		NIHSS ≥ 6	113	0.00	0.75	0.162	0.108
		Missing	7	0.11	0.29	0.179	0.063
		Total	1316	0.00	0.75	0.191	0.121

Partitioning the sample and running separate regressions for the NIHSS = 0–5 ('low severity') and NIHSS ≥ 6 ('moderate to high severity') subgroups produced an improvement in model fit and predictive validity for both index- and item-based Barthel algorithms. Parameter estimates and model fit for the index- and item-based 'severity-specific' Barthel algorithms are given in Table 8. Post-sample tests of predictive validity for the index- and item-based 'severity-specific' Barthel algorithms are reported in Table 10. Despite these improvements, comparison between mean predicted and mean observed AQoL utility scores implies that the predictive validity of the index- and item-based Barthel algorithms remains inadequate for the purposes of economic evaluation across the full range of stroke severity. Predicted and observed AQoL scores were significantly different for the item-based Barthel algorithm in the NIHSS = 0 (t = 2.040, p = 0.041) and NIHSS = 1–5 (t = -2.625, p = 0.009) subgroups but not in the NIHSS ≥ 6 subgroup (t = -0.360, p = 0.719), even when the 'low severity' algorithm was used to predict AQoL scores for NIHSS = 0 and NIHSS = 1–5 subgroups, and the 'moderate to severe' algorithm was used to predict AQoL scores for the NIHSS ≥ 6 subgroup.

**Table 10:** Post-sample predictive validity for Barthel 'severity-specific' algorithms

Model	Criteria	Group	N	Min	Max	Mean	SD
Observed AQoL	Validation sample	NIHSS = 0	844	-0.04	1.00	0.536	0.334
		NIHSS = 1–5	352	-0.04	1.00	0.446	0.299
		NIHSS ≥ 6	113	-0.04	0.98	0.111	0.199

Predicted AQoL	Index-based	NIHSS = 0*	844	0.00	0.68	0.514	0.229
		NIHSS = 1–5*	352	0.00	0.68	0.483	0.230
		NIHSS ≥ 6^	113	0.00	0.56	0.123	0.166
	
	Item-based	NIHSS = 0*	844	0.13	0.62	0.510	0.160
		NIHSS = 1–5*	352	0.13	0.62	0.493	0.153
		NIHSS ≥ 6^	113	-0.01	0.60	0.120	0.176

Mean Absolute Deviation (MAD)	Index-based	NIHSS = 0*	844	0.00	0.66	0.167	0.115
		NIHSS = 1–5*	352	0.00	0.74	0.182	0.142
		NIHSS ≥ 6^	113	0.00	0.91	0.097	0.131
	
	Item-based	NIHSS = 0*	844	0.00	0.60	0.196	0.117
		NIHSS = 1–5*	352	0.00	0.60	0.193	0.130
		NIHSS ≥ 6^	113	0.00	0.89	0.090	0.128

*Predicted values obtained from 'low severity' algorithm. ^Predicted values obtained from 'moderate to severe severity' algorithm

While mean predicted AQoL utility scores from the index-based severity-specific Barthel algorithms were not *significantly *different from their corresponding mean observed scores at the 0.05 level in NIHSS = 0 (t = 1.578, p = 0.115), NIHSS = 1–5 (t = -1.840, p = 0.066) and NIHSS ≥ 6 subgroup (t = -0.360, p = 0.719) subgroups, differences approaching *clinical *significance were observed for the NIHSS = 1–5 subgroup. The difference between mean predicted and mean observed scores in the NIHSS = 1–5 subgroup approached 0.04 (95%CI:0.00–0.08) – a magnitude of error that could potentially mask between-group or pre-post treatment effects. While there may be circumstances where the expected treatment effects from stroke interventions are detectable even in the presence of upper bound errors associated with predicted scores, the Barthel algorithm described above will not always produce 'conservative' estimates. Note, for example, that the item-based algorithms underestimate the mean observed AQoL score for the NIHSS = 0 subgroup but provide an overestimate for the NIHSS = 1–5 subgroup. Where conversion algorithms have the potential to make interventions appear more cost-effective and push borderline interventions under the funding threshold, the use of predicted scores from such algorithms is unlikely to be acceptable to decision-makers.

## Discussion

Previous applications of the TTU approach have demonstrated the feasibility and value of regression-based transformations for deriving QALY-weights from *generic *descriptive measures of health and HRQoL [6-10]. For example, a number of generic to generic transformations from the SF-36/-12 to preference-based measures have recently been validated in a sample of patients at risk of stroke [27] and in post-stroke patients [28].

Pickard et al. [28] predicted QALY-weights by applying patient-level data to the Brazier et al. [29] SF-36-based SF6D algorithm, the Brazier and Roberts [30] SF-12-based SF6D algorithm and several of the SF-36/-12-based TTU regression-based algorithms [6-8,31-34] reviewed elsewhere [9]. The study sample for the Pickard et al. [28] validation study included 81 of the 124 patients with confirmed ischaemic stroke enrolled in a longitudinal study of post-stroke HRQoL [35] for whom observations were available on the SF-36 at baseline and follow-up. While Pickard et al. did not provide a comparison between predicted and observed QALY-weights, their comparison of incremental cost-utility ratios (ICURs) derived using different conversion algorithms provided a test of convergent validity. Pickard et al. reported a three-fold difference in ICURs derived from different algorithms and concluded that "...the choice of algorithm could determine whether the intervention is considered cost-effective or unacceptable" (p6).

Kaplan et al. [27] derived QALY-weights from patient-level SF-36 data using the Brazier et al. [36] SF-36-based SF6D and the Fryback [7] and Nichol [33] SF-36/-12-based TTU regression algorithms. The study sample for the Kaplan et al. [27] validation study included 294 patients at risk of stroke from the Quality of Life in Stroke Prevention (QLASP) study. Kaplan et al. [27] reported a strong correlation between predicted QALY-weights from the Brazier [36], Fryback [7] and Nichol [33] algorithms but a sometimes modest correlation between predicted and observed QALY-weights. Kaplan et al. [27] concluded that conversion algorithms produced comparable, but not interchangeable results.

Against the background of this previous research, we have conducted the first study to derive and validate conversion algorithms in a sample of stroke patients for multiple stroke-relevant outcome measures. Our findings can be summarised as follows. For the item- and subscale-based SF-36 algorithms, differences between mean predicted and mean observed AQoL scores were neither clinically nor statistically significant when the 'low severity' algorithm was used to predict AQoL scores for patients in the NIHSS = 0 and NIHSS = 1–5 subgroups and the 'moderate to severe severity' algorithm was used to predict AQoL scores for patients in the NIHSS ≥ 6 subgroup. Model fit and predictive power for our final generic (SF-36) to generic (AQoL) regression-based transformation were superior when compared to TTU regressions included in previous validation studies conducted in stroke patients [27,28]. The superior explanatory power of our transformations may be attributable to a better correspondence between the coverage of the SF-36 and the AQoL than between the SF-36 and other preference-based measures such as the EQ5D, HUI2/3 or the QWB. Hawthorne, Richardson and Day [13] concluded that coverage of the HRQoL universe was poor for the QWB but good or very good for the HUI2 and AQoL. It might also be the case a lower noise (random variation) to signal (systematic variation) ratio in the AQoL as compared to the HUI2 or QWB might increase the share of variation that can be explained; simply because there is less random error to be discarded as a residual. Whatever the reason, our findings suggest that the predictive validity of our severity-specific item-based and subscale-based SF-36 to AQoL algorithms is more than adequate for evaluating the relative effectiveness and cost-effectiveness of stroke interventions.

With regards to our disease-specific to generic transformations, the difference between mean predicted and mean observed AQoL scores from the NIHSS algorithms reached clinical and statistical significance in at least one subgroup for all models. The relatively poor predictive power of our NIHSS to AQoL transformations is not surprising given the differences in sensitivity and coverage between the NIHSS and the AQoL. Transformation of the NIHSS scale to the AQoL requires mapping from a detailed description of a relatively narrow area of HRQoL space to a much more general description covering multiple dimensions of HRQoL. Variation in AQoL scores for stroke patients might arise due to variation in emotional well-being, physical senses, self-care, household tasks and/or mobility such that it is difficult to see how the NIHSS scales could closely approximate stroke outcomes along the AQoL scale. For disease-specific measures that are designed to provide a detailed picture of only one of several potentially relevant dimensions or that cover different dimensions than the preference-based 'target' instrument, TTU regression is unlikely to provide a satisfactory transformation.

For the 'moderate to severe' index- and item-based Barthel to AQoL algorithm, differences between mean predicted and mean observed AQoL scores were neither clinically nor statistically significant for patients in the NIHSS ≥ 6 subgroup. While the 'severity-specific' Barthel to AQoL algorithms therefore represent a substantial improvement on the NIHSS to AQoL algorithms, it remains the case that differences between predicted and observed AQoL scores from the Barthel algorithms reached levels that could potentially mask minimally important differences *over some segments of the severity scale*. When the low-severity index-based Barthel algorithm was used to predict AQoL scores for the NIHSS = 1–5 subgroup, the difference between mean predicted and mean observed scores approached 0.04 (95%CI:0.00–0.08) – a magnitude of error that could be considered clinically significant and potentially unacceptable to decision-makers. Analysts and policy-makers should therefore exercise caution when using predicted scores from our severity-specific Barthel to AQoL algorithms *in samples that include low severity patients*. The predictive validity of our moderate to severe Barthel to AQoL algorithm should, however, be adequate for the purposes of evaluating the relative effectiveness and cost-effectiveness of stroke interventions in patients with moderate to severe stroke severity.

While the predictive validity for several of the regression-based mappings described above appear to be acceptable for predicting between-group differences, our findings are subject to a number of limitations. It should, for example, be emphasised that none of our mappings were deemed suitable for the purposes of predicting health state utilities or change scores *at the individual level*. To the extent that the coverage and sensitivity of the descriptive and preference-based measures diverge, residual error (potentially precluding the sort of precision required for prediction at the individual level) is unavoidable in a 'self-contained' mapping that would permit SF-36, Barthel or NIHSS data to be converted to AQoL utility scores without relying on additional data that may or may not be available in a particular application.

It should also be emphasised that use of our severity-specific algorithms requires some means of distinguishing 'low severity' patients (whose AQoL scores are most appropriately estimated using the 'low severity' algorithms) from 'moderate to high' severity patients (whose AQoL scores are most appropriately estimated using the 'moderate to severe' algorithms). During estimation, we used the NIHSS to partition the sample into 'low' and 'moderate to high' severity subgroups and the end-user could make similar reference to NIHSS scores in assigned patients or samples to the appropriate algorithm. This is, of course, contingent on the availability of NIHSS data to the end-user in the relevant dataset. It could therefore be argued that using the SF-36 rather than NIHSS to partition the sample during estimation would have made the severity-specific SF-36 to AQoL algorithms more useful and less reliant on additional data. Likewise, it could be argued that using the Barthel rather than NIHSS to partition the relevant estimation sample would have made the severity-specific Barthel algorithms more 'self-contained'. Such arguments would carry particular weight where the derived transformation algorithms are intended for use across multiple conditions. This is not, however, the case in the present study where the intention was to derive algorithms specifically designed for use in stroke. Given the available data, the NIHSS provided a convenient way of identifying clinically distinct groups of patients but it should also be possible to identify low severity and moderate to high severity stroke patients based on clinical assessment (rather than relying on the availability of NIHSS data). Further validation studies will, however, be required to confirm that our 'severity-specific' algorithms are applicable in samples partitioned using clinical assessment.

For the present study, we chose between fixed and random effects models using a Hausman specification test [[20], p576]; with fixed effects frequently identified as our preferred model. However, it is sometimes argued that the random effects model is to be preferred whenever results will be used to draw inferences regarding the distribution of a wider population [37]. Greene [20] offers a different perspective, noting that arguments in favour of fixed or random effects frequently fail to provide unambiguous guidance; and concludes that the choice between fixed and random effects should instead be driven by the data. Specifically, the random effects model treats the cluster-specific effects as uncorrelated with other regressors and, where this assumption is not supported by the data, the random effects model will suffer from inconsistency due to omitted variables and should be rejected [20]. In this context, it is worth emphasizing that interpretation of our findings should respect the assumptions and limitations of the models used in estimation.

Finally, it should be emphasised that the models estimated in the present study are not intended for application in non-stroke populations. The weight attached to each item, subscale or scale entering each of our conversion algorithms reflects the covariance in our data between AQoL health states and Barthel, NIHSS or SF-36 health states. Because this covariance is likely to be quite different in stroke than in other disease-areas or the general population, our conversion algorithms may not be applicable to non-stroke populations. More generally, our findings are contingent upon the characteristics of our study population and on the coverage and sensitivity of the descriptive and preference-based measures used to generate our conversion algorithms. Note, for example, that our findings regarding the feasibility and value of TTU regression in stroke-specific outcome measures might not be generalisable to all condition-specific measures in all disease-areas. Likewise, where transformations have been derived and validated in a sample of stroke-patients with mean age exceeding 70 years, those transformations cannot be assumed valid for the purposes of predicting QALY-weights *in children with stroke*.

Despite these limitations, the conversion algorithms reported here represent an improvement on the regression-based conversion algorithms that have previously been validated for use in stroke [27,28]. Moreover, our derivation of a Barthel to AQoL transformation for moderate to severe stroke widens the set of descriptive stroke-specific measures that can be transformed to obtain preference-based outcomes suitable for use in economic evaluation. The present study therefore adds additional tools to the analyst's tool-box; increasing the chances that an appropriate tool with be available for the job at hand. Findings from the present study also provide a unique insight into the feasibility and value of TTU regression in stroke-specific outcome measures such as the Barthel and NIHSS; highlighting the necessity of some minimal correspondence between the condition-specific 'base' measure and the preference-based 'target' with respect to coverage and sensitivity.

## Conclusion

Our findings suggest that TTU regression can provide a useful second-best approach for deriving QALY-weights associated with stroke disease-states. While the NIHSS to AQoL transformations proved unsuitable for most applications, transformations from the SF-36 and Barthel to the AQoL provided sufficient predictive power to suggest that stroke-relevant outcomes can be transformed to preference-based measures for the purposes of economic evaluation. While a number of generic to generic transformations from the SF-36 to preference-based outcome measures are now available, the SF-36 to AQoL transformations reported here are the only published transformations to have been derived and validated in a sample of stroke patients [9]. Moreover, our derivation of a Barthel to AQoL transformation for moderate to severe stroke widens the set of descriptive stroke-specific measures that can be transformed for use in economic evaluation. Our findings also suggest that attempts to derive regression-based algorithms from stroke-specific descriptive measures such as the NIHSS to generic preference-based measures such as the AQoL will sometimes be frustrated by a lack of correspondence in the sensitivity and/or coverage of 'base' and 'target' instruments.

The implications for practice are two-fold. First, it is anticipated that our transformations will prove to be a valuable tool for analysts and should allow the best use to be made of the available data; improving the quality and policy-relevance of economic evaluations for stroke interventions. Second, improvements in the economic evaluation of stroke interventions should allow clinicians and policy-makers to make better decisions; potentially saving money and improving patient outcomes. Our findings also have a number of implications for research. First, researchers may wish to take account of the feasibility of TTU regression in certain condition-specific measures (but not in others) when selecting descriptive outcome measures for inclusion in clinical trials. Such considerations will be particularly important where resource constraints or patient burden preclude the direct observation of preference-based measures in the trial population. Second, researchers attempting to derive their own regression-based transformations for other descriptive measures should take particular note of the improvements in predictive validity that we were able to obtain by deriving separate transformations for clinically distinct subgroups of patients. Finally, our findings suggest that validity in predicting group-wise differences will not always translate to validity in predicting health state utilities or change scores for individual patients. Researchers responsible for the derivation of regression-based transformations might therefore wish to provide guidelines for end-users to ensure use consistent with validation data.

## Competing interests

The authors declare that they have no competing interests

## Authors' contributions

DM participated in the design of the study, data analysis and interpretation of results, and drafted the manuscript. LS participated in the design of the study and interpretation of results, and suggested edits and revisions to the manuscript. JS contributed to the acquisition and interpretation of the data, participated in the interpretation of results, and suggested edits and revisions to the manuscript. All authors read and approved the final manuscript.

## References

[B1] Richardson J (1994). Cost utility analysis: What should be measured?. Social Science and Medicine.

[B2] Gold M, Siegel J, Russell L, Weistein MC (1996). Cost-effectiveness in health and medicine.

[B3] Bansback NJ, Regier DA, Ara R, Brennan A, K. S, Esdaile JM, Anis AH, Marra CA (2005). An overview of economic evaluations for drugs used in rheumatoid stroke – Focus on tumour necrosis factor-alpha antagonists. Drugs.

[B4] Neumann PJ, Goldie SJ, Weinstein MC (2000). Preference-based measures in economic evaluation in health care. Annual Review of Public Health.

[B5] Segal L, Day SE, Chapman AB, Osborne RH (2004). Can we reduce the burden from osteoarthritis? An evidence-based priority-setting model. Medical Journal of Australia.

[B6] Franks P, Lubetkin EI, Gold MR, Tancredi DJ, Jia H (2004). Mapping the SF-12 to the EuroQol EQ-5D Index in a national US sample. Medical Decision Making.

[B7] Fryback DG, Lawrence WF, Martin PA, Klein R, Klein BE (1997). Predicting quality of well-being scores from the SF-36: Results from the Beaver Dam Health Outcomes Study. Medical Decision Making.

[B8] Lawrence WF, Fleishman JA (2004). Predicting EuroQoL EQ-5D preference scores from the SF-12 health survey in a nationally representative sample. Medical Decision Making.

[B9] Mortimer D, Segal L (2008). Comparing the incomparable? A systematic review of competing techniques for mapping one health outcome measure into another. Medical Decision Making.

[B10] Mortimer D, Segal L, Hawthorne G, Harris A (2007). Item-based versus scale-based mappings from the SF-36 to a preference-based quality of life measure. Value in Health.

[B11] Thrift AG, Dewey HM, Macdonnell RA, McNeil JJ, Donnan GA (2000). Stroke incidence on the east coast of Australia: the North East Melbourne Stroke Incidence Study (NEMESIS). Stroke.

[B12] Hatano S (1976). Experience from a multicentre stroke register: a preliminary report. Bulletin of the World Health Organization.

[B13] Hawthorne G, Richardson J, Day N (2001). A comparison of the Assessment of Quality of Life (AQoL) with four other generic utility instruments. Annals of Medicine.

[B14] Hawthorne G, Richardson J, Osborne R (1999). The Assessment of Quality of Life (AQoL) Instrument: a psychometric measure of health related quality of life. Quality of Life Research.

[B15] Sturm JW, Osborne RH, Dewey HM, Donnan GA, Macdonnell RA, Thrift AG (2002). Brief comprehensive assessment of quality of life after stroke: the Assessment of Quality of LIfe (AQWoL) instrument in the North East Melbourne Stroke Incidence Study (NEMESIS). Stroke.

[B16] Ware J (2000). Using generic measures of functional health and well-being to increase understanding of disease burden. Spine.

[B17] Ware J, Kosinski M, Keller S (1994). SF-36 physical and mental health summary scales: A user's manual.

[B18] Brott T, Adams HP, Olinger CP, Marler JR, Barson WG, Biller J, Spilker J, Holleran R, Eberle R, Hertzberg V (1989). Measurments of acute cerebral infarction: a clinical examination scale. Stroke.

[B19] Mahoney F, Barthel D (1965). Functional evaluation: the Barthel Index. Maryland Medical Journal.

[B20] Greene WH (1993). Econometric Analysis.

[B21] Goldstein H, Browne W, Rasbash J (2002). Partitioning variation in multilevel models. Understanding Statistics.

[B22] Harvey AC (1981). The Econometric Analysis of Time Series.

[B23] O'Brien BJ, Spath M, Blackhouse G, Severens JL, Dorian P, Brazier J (2003). A view from the bridge: agreement between the SF-6D utility algorithm and the Health Utilities Index. Health Economics.

[B24] (2006). SPSS 15.0 for Windows.

[B25] (2005). STATA/SE Version 8.2 for Windows.

[B26] Hawthorne G, Osborne R (2005). Population norms and meaningful differences for the Assessment of Quality of Life (AQoL) measure. Australian and New Zealand Journal of Public Health.

[B27] Kaplan RM, David K, Ganiats TG (2002). Comparison between three methods for imputing utility scores from the SF-36. Presented at 9th Annual Conference of the International Society for Quality of Life Research (ISOQOL).

[B28] Pickard AS, Wang Z, Walton SM, Lee TA (2005). Are decisions using cost-utility analyses robust to choice of SF-36/SF-12 preference-based algorithm?. Health & Quality of Life Outcomes.

[B29] Brazier J, Roberts J, Deverill M (2002). The estimation of a preference-based measure from the SF-36. Journal of Health Economics.

[B30] Brazier J, Roberts J (2004). The Estimation of a Preference-Based Measure of Health from the SF-12. Medical Care.

[B31] Franks P, Lubetkin EI, Gold MR, Tancredi DJ (2003). Mapping the SF-12 to preference-based instruments – Convergent validity in a low-income, minority population. Medical Care.

[B32] Lundberg L, Johannesson M, Isacson DGL, Borgquist L (1999). The relationship between health-state utilities and the SF12 in a general population. Medical Decision Making.

[B33] Nichol MB, Sengupta N, Globe D (2001). Evaluating quality-adjusted life years: Estimation of the HUI2 from the SF-36. Medical Decision Making.

[B34] Shmueli A (2004). The relationship between the visual analogue scale and the SF-36 scales in the general population: An update. Medical Decision Making.

[B35] Pickard A, Johnson JA, Feeny DH, Carriere KC, Shuarib A, Nasser AM (2004). Agreement between patient and proxy assessments of health-related quality of life after stroke using the EQ5D and Health Utilities Index. Stroke.

[B36] Brazier J, Usherwood T, Harper R, Thomas K (1998). Deriving a preference-based single index from the UK SF-36 health survey. Journal of Clinical Epidemiology.

[B37] Gelman A, Hill J (2007). Data Analysis Using Regression and Multilevel/Hierarchical Models.

